# Targeting ligand-dependent wnt pathway dysregulation in gastrointestinal cancers through porcupine inhibition

**DOI:** 10.1016/j.pharmthera.2022.108179

**Published:** 2022-10

**Authors:** Dustin J. Flanagan, Simon A. Woodcock, Caroline Phillips, Catherine Eagle, Owen J. Sansom

**Affiliations:** a Cancer Research UK Beatson Institute, Glasgow, UK; b Biomedicine Discovery Institute, Monash University, Melbourne, Australia; c Redx Oncology Ltd, Redx Pharma PLC, UK; d Institute of Cancer Sciences, University of Glasgow, Glasgow, UK

**Keywords:** Porcupine inhibitor, Wnt, gastrointestinal cancer, RNF43, RSPO, clinical trials, APC, adenomatous polyposis coli, CAF, cancer associated fibroblast, CMS, consensus molecular subtype, CRC, colorectal cancer, CSC, cancer stem cell, EMT, epithelial-mesenchymal transition, Dkk, Dickkopf, HCC, hepatocellular carcinoma, LGR, leucine-repeat G-coupled receptor, LRP, low-density lipoprotein receptor-related protein, MSI, microsatellite instability, PCP, planar cell polarity, ROR, receptor tyrosine kinase-like orphan receptors, RSPO, R-spondin, SFRP, Secreted frizzled-related protein, TP53, transformation-related protein 53

## Abstract

Gastrointestinal cancers are responsible for more cancer deaths than any other system of the body. This review summarises how Wnt pathway dysregulation contributes to the development of the most common gastrointestinal cancers, with a particular focus on the nature and frequency of upstream pathway aberrations. Tumors with upstream aberrations maintain a dependency on the presence of functional Wnt ligand, and are predicted to be tractable to inhibitors of Porcupine, an enzyme that plays a key role in Wnt secretion. We summarise available pre-clinical efficacy data from Porcupine inhibitors *in vitro* and *in vivo*, as well as potential toxicities and the data from early phase clinical trials. We appraise the rationale for biomarker-defined targeted approaches, as well as outlining future opportunities for combination with other therapeutics.

## 1 Introduction

Secreted Wnt proteins were originally discovered as key players in development and oncogenesis (Nusse & Varmus, 1982; Nüsslein-Volhard & Wieschaus, 1980; Sharma & Chopra, 1976) and subsequently found to be important in tissue homeostasis, with pivotal roles in stem cell-mediated regeneration of the intestinal epithelium and maintenance of bone integrity (Clevers & Nusse, 2012) Dysregulation of Wnt signaling has been implicated in various cancers, particularly those of the gastrointestinal tract (Jackstadt, Hodder, & Sansom, 2020; Sphyris, Hodder, & Sansom, 2021).

**Fig. 1:**
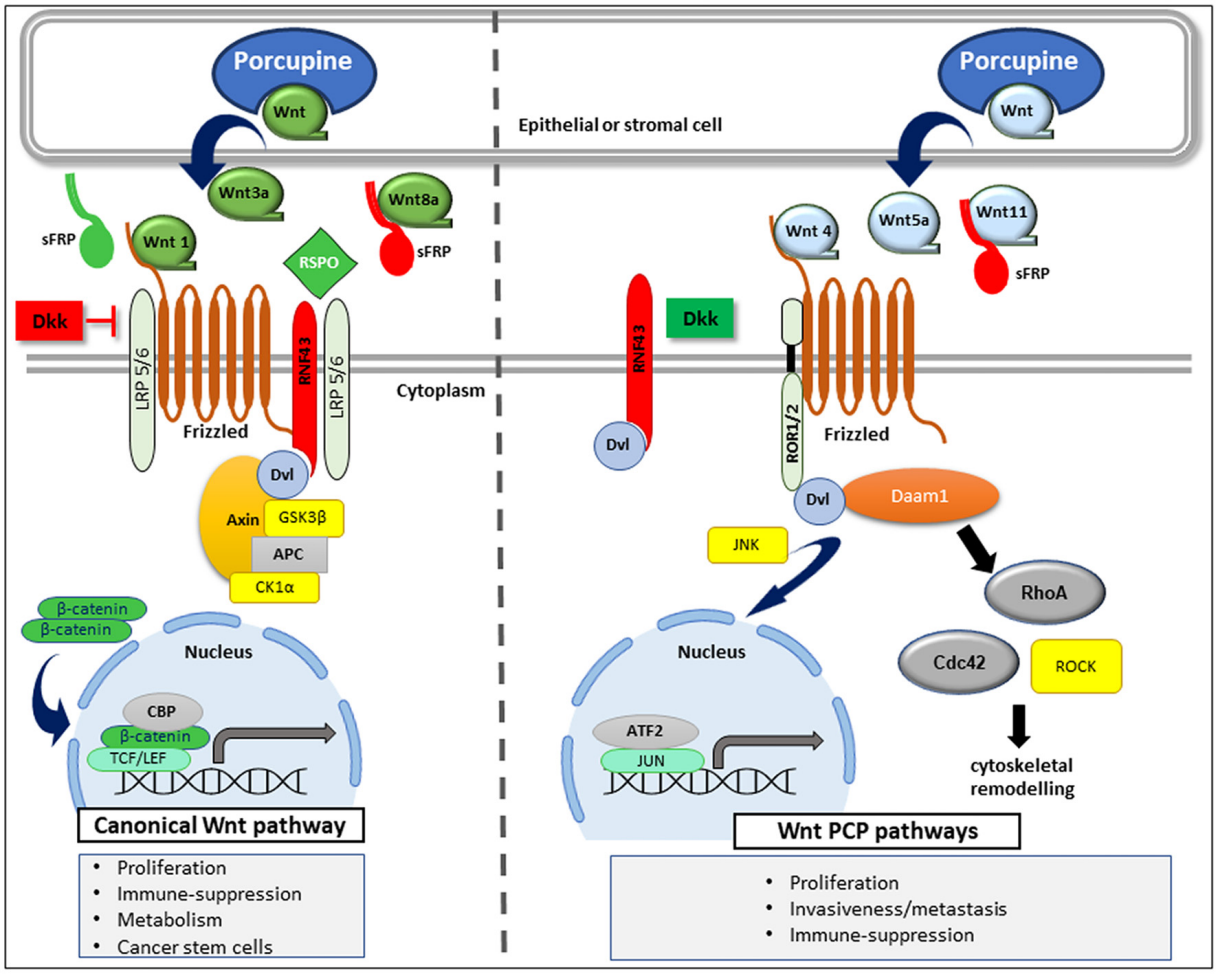
Canonical and Non-canonical Wnt Signaling in Cancer Both canonical and non-canonical Wnt ligands require Porcupine activity for secretion. Canonical Wnts bind Frizzled receptors in complex with LRP5/6 to activate downstream signaling by phosphorylation and stabilization of β-catenin, which enters the nucleus to activate gene transcript via TCF/LEFs. Non-canonical Wnts also act via Frizzled receptors, activating ROR1/2 or recruiting Daam1 to activate the planar cell polarity (PCP) pathways, which can activate gene transcription or promote actin cytoskeleton rearrangements. Both pathways are subject to extracellular regulation, with R-spondins (RSPO) potentiating canonical signaling, and the ubiquitin-ligase RNF43 (or its close relative ZNRF3) and Dkk acting as negative regulators (depicted in red). sFRPs may act as positive or negative regulators of canonical signalling depending on context. sFRPs and RNF43 can also regulate non-canonical signaling, although Dkks have been shown to activate PCP pathways, in one of several examples of cross-talk between different Wnt pathways. LRP: low-density lipoprotein receptor-related protein, APC: adenenomatous polyposis coli, GSK3β: Glycogen synthase kinase 3β, CK1α: casein kinase 1α, Daam1: dishevelled associated activator of morphogenesis 1, Dvl: dishevelled; ROR1/2, receptor-tyrosine kinase-like orphan receptor 1/2, TCF/LEF: transcription factor/lymphoid enhancer binding factors, sFRP: secreted frizzled-related protein, Dkk; Dikkopf.

Extracellular Wnts can be divided into canonical or non-canonical according to the cellular pathways they activate (Fig. 1). Canonical Wnts bind Frizzled receptors which, in complex with low-density lipoprotein receptor-related protein (LRP) 5 or 6 and Dishevelled, release β-catenin from a destruction complex of Axin, adenomatous polyposis coli (APC), casein kinase 1α (CK1α) and glycogen synthase kinase 3β (GSK3β). This permits accumulation of β-catenin and its entry into the nucleus to initiate activation of target genes in combination with transcription factor/lymphoid enhancer binding factors (TCF/LEF) (Clevers & Nusse, 2012). The pathway is subject to several checks and balances, particularly in the extracellular space (Niehrs, 2012). The transmembrane E3 ligase RNF43 and its close relative, ZNRF3, negatively regulate canonical Wnt signaling through ubiquitylation of Frizzled receptors, which targets them to lysosomes for destruction (Koo et al., 2012). Soluble Dickkopf (Dkk) proteins compete with Wnt binding to LRP5/6 (Kagey & He, 2017) to block canonical signaling. Wnt activation can be fine-tuned by extracellular R-spondins (RSPOs), which bind to leucine-repeat G-coupled receptors (LGR/5/6) on the cell surface, bringing RSPO into contact with RNF43/ZNRF3. This interaction leads to membrane clearance of RNF43/ZNRF3 with a concomitant increase in membrane Frizzled receptors, thus enhancing Wnt signalling (De Lau, Peng, Gros, & Clevers, 2021). Secreted frizzled-related proteins (SFRPs) can sequester Wnts and prevent them binding Frizzled receptors, although they have also been reported to act as agonists, depending on concentration and/or context (Routledge & Scholpp, 2019; Xavier et al., 2014).

Pathways not involving β-catenin are termed non-canonical. One of the most well understood is the planar cell polarity (PCP) pathway, in which non-canonical Wnts such as Wnt5a activate Frizzled and co-receptors such as tyrosine kinase-like orphan receptors (RORs) to activate gene expression via various transcription factors through Jun N-terminal kinase (JNK) activation, or promote rearrangements of the cytoskeleton via dishevelled associated activator of morphogenesis 1 (Daam1) which activates small GTPases such as Cdc42 and RhoA (Mattes et al., 2018; Menck, Heinrichs, Baden, & Bleckmann, 2021). Non-canonical Wnts are also subject to direct negative regulation by SFRPs (Satoh, Matsuyama, Takemura, Aizawa, & Shimono, 2008) and RNF43 (Radaszkiewicz et al., 2021; Tsukiyama et al., 2015) but not Dkks, which exclusively and negatively regulate canonical signaling via LRP5/6. Indeed, Dkks have been suggested to activate non-canonical signaling in a non-direct manner, possibly by releasing Frizzled receptors from LRP5/6 complexes, thereby making them available for non-canonical Wnts (Niehrs, 2006). Dkk1 may also function via other receptors, such as cytoskeleton-associated protein 4, an interaction which may be of relevance in pancreatic and lung cancer (H. Kimura et al., 2016). Cross-talk between Wnt pathways is common (Topol et al., 2003), and it has been proposed that Wnt signaling should be viewed as a complex, integrated network, rather than a linear pathway (Amerongen & Nusse, 2009; Menck et al., 2021).

## 2 Dysregulation of Wnt Pathways in Gastrointestinal Cancers

Elevated β-catenin, indicative of canonical Wnt pathway activation, has been observed in many gastrointestinal tumors. Such activation may be driven by mutations in upstream pathway components such as *RNF43* and *RSPO* fusions and tumors will remain dependent on Wnt ligand, or mutations in downstream Wnt pathway components such as *APC* or *CTNNB1*, the gene encoding β-catenin, where the pathway maybe activated independent of Wnt ligand. Deregulation of extracellular Wnt regulators such as Dkks and SFRPs via epigenetic mechanisms may also play a role (for reviews see e.g (Kleeman & Leedham, 2020; White, Chien, & Dawson, 2012)). Activation of the non-canonical pathway in cancers has also been associated with poor prognosis (Chen, Chen, Tang, & Xiao, 2021). Although proliferation and stem cell regeneration have been identified as potential cancer-promoting mechanisms, Wnt pathways also promote tumor evasion of host immunity (Luke, Bao, Sweis, Spranger, & Gajewski, 2019; Oderup, Lajevic, & Butcher, 2021; Valencia et al., 2011; Wang, Kalland, Ke, & Qu, 2018), as well as increasing migration and invasiveness via epithelial-mesenchymal transition (EMT) (Hlubek et al., 2007). These findings have led to a search for therapeutics targeting Wnt pathways. One of several emerging drug candidates is the membrane-bound protein-serine O-palmitoleoyltransferase Porcupine, encoded by the *PORCN* gene. Porcupine is located in the endoplasmic reticulum of stromal or epithelial cells and palmitoylates Wnt ligands, an enzymatic process essential for their secretion and binding to Frizzled receptors (Liu et al., 2013). Since Porcupine targets both canonical and non-canonical Wnts (Madan et al., 2016), inhibitors have the potential to hit multiple pro-tumorigenic mechanisms. However, they will only block signaling that remains dependent on secreted Wnts. In the next section, we summarise evidence for the involvement of Wnt pathway aberrations in five leading gastrointestinal cancers, with a focus on ligand-dependent mechanisms.

## 3 Wnt pathway dysregulation in colorectal cancer (CRC)

**Fig. 2:**
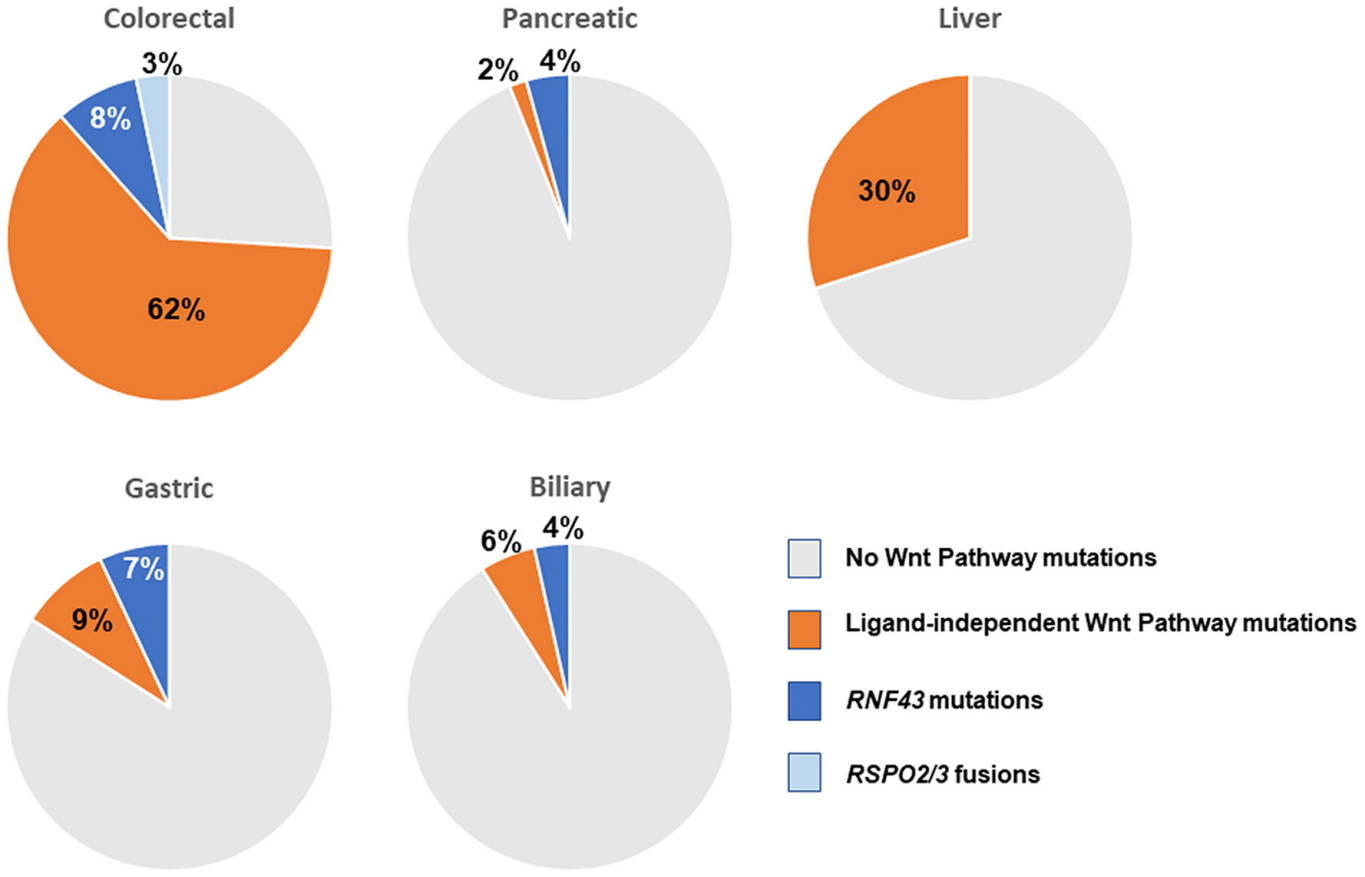
Contribution of Genetic Aberrations in Wnt Pathways in Gastrointestinal Cancers Mutations or fusions in Wnt pathway genes are common in gastrointestinal cancers. Of these, the majority occur in downstream components such as *CTNNB1* and *APC*, which are expected to be largely independent of extracellular Wnt ligands, although ligand-dependent aberrations such as *RNF43* mutations or *RSPO2/3* fusions are also observed. Mutation data from cBio Portal, (Cerami et al., 2012; Gao et al., 2013), using curated non-overlapping datasets, n = 1564 samples for CRC, n = 922 for pancreatic cancer, n = 1111 for liver cancer, n = 795 for gastric cancer, n = 588 for biliary cancer. CBio Portal accessed 27^th^ Oct 2021. Since *RSPO2/3* fusions are not captured in cBio, prevalence is per literature reports (Kleeman et al., 2020).

CRC is a Wnt-pathway driven disease, with 63-93% of patients having mutations in at least one component of the canonical Wnt pathway (Fig. 2) (Cancer Genome Atlas Network, 2012; Kleeman et al., 2020; Yaeger et al., 2018). The majority of mutations occur in downstream elements of the pathway. Most common are loss of function mutations in *APC*, which account for 60-80% of Wnt pathway aberrations in colorectal cancers (Kinzler & Vogelstein, 1996; Kleeman et al., 2020; Yaeger et al., 2018), and to a lesser extent, aberrations in other downstream components *AXIN1*, *AXIN2*, and *CTNNB1*. In *APC*-mutated cancer models, restoration of functional APC protein is effective in reversing tumorigenesis (Dow et al., 2015; Faux et al., 2004; Zilberberg, Lahav, & Rosin-Arbesfeld, 2010), but with current technologies, APC restoration is not a practical therapeutic option. In theory, cancers driven by such downstream activation mechanisms are unlikely to respond to Porcupine inhibition, as they do not depend on Wnt ligand, and are therefore termed ligand-independent. However, there are reports of APC-deficient and *CTNNB1*-mutant colorectal cell lines that remain dependent on Wnt ligands, and whose proliferation was reduced by Porcupine inhibition (J. Li, Zhang, Wang, & Zhang, 2019; Voloshanenko et al., 2013). Whether the dependency of *APC*- or *CTNNB1*-mutant CRC cells on Wnt extends beyond *in vitro* models requires further investigation.

10-20% of CRC patients have pathway aberrations in which a requirement for Wnt ligand is maintained. The majority of these are in *RNF43* or *ZNRF3*, with *RNF43* mutations found in 9-18% of CRC patients, most of which associate with microsatellite instability and are mutually exclusive from genetic aberrations in *APC* but strongly associated with *BRAF* mutations (Giannakis et al., 2014; Yaeger et al., 2018). Many *RNF43* mutations observed in CRC are truncations and hence loss of function, and therefore predicted to activate both canonical and non-canonical Wnt signaling. Certain truncation mutants have been reported to switch the protein from a tumor suppressor into an activator of downstream β-catenin signaling (Spit et al., 2020). Growth of tumorigenic intestinal organoids that lack functional RNF43 or ZNRF3 remain dependent on local Wnt ligand production (Koo, Van Es, Van Den Born, & Clevers, 2015), and their growth can be blocked by Porcupine inhibitors (Van De Wetering et al., 2015). However, there is debate concerning the most common *RNF43* mutation, G659Vfs*41, a frameshift mutation which is almost exclusively found in microsatellite instability (MSI) patients. Initial reports suggested it was associated with cancer cell growth and/or survival, but it has also been reported to generate fully functional protein, although potentially one that is less stable than wild-type and associated with lower RNF43 expression (Giannakis et al., 2014; Li et al., 2020; Tu et al., 2019; Yu et al., 2020). Oncogenic effects of this mutation have been reported to be independent of Wnt signaling (L. Fang et al., 2021). It is therefore unclear whether tumors with this particular frameshift retain Wnt ligand dependency in patients or in fact have enhanced Wnt pathway signalling as a result of the mutation.

*RSPO2* and *RSPO3* fusions are mutually exclusive and are present in 2-10% of CRC, co-occurring with *KRAS* or *BRAF* mutations, but only very rarely with *APC* and mutually exclusive of *CTNNB1* and *RNF43* mutations (Giannakis et al., 2014; Seshagiri et al., 2012; Shinmura et al., 2014). The gene encoding *RSPO3* fuses with the signal sequence and early section of the *PTPRK* gene leading to increased expression of RSPO3 and activation of the canonical Wnt pathway. Genetic fusions of *RSPO2* have been observed with *EIF3E* and *PIEZO1,* and these were associated with increased RSPO2 expression (Hashimoto et al., 2019). Increased stromal RSPO3 expression has also been observed in the absence of gene fusions and this stromal overexpression of RSPO3 is strongly associated with the aggressive mesenchymal consensus molecular subtype 4 (CMS4) CRC phenotype (Kleeman et al., 2020). Targeting RSPO3 overexpression to the murine gut epithelium upregulates Wnt signaling and drives the formation of ectopic niche compartments and intestinal stem cells, leading to adenoma lesions (Hilkens et al., 2017). Inhibition of RSPO3 with function-blocking antibodies in *PTPRK-RSPO3*-fusion tumor xenografts inhibited growth and promoted epithelial differentiation (Storm et al., 2016).

Excessive Wnt signaling can also arise by non-mutational mechanisms. For example, epigenetic silencing of negative Wnt regulators has been observed during CRC cancer progression and was associated with increased nuclear β-catenin (Caldwell et al., 2004, Silva et al., 2014, Suzuki et al., 2004, Zhang et al., 2008). Epigenetic suppression of Wnt feedback loops, including reduced Axin2 expression, has been observed in CRC and low Axin2 expression has been postulated to be a discriminatory biomarker for ligand-dependent disease (Kleeman et al., 2020). However, the expression of some negative Wnt regulators (Notum, Wif1, Dkk3) is very low/absent in normal murine gut tissue (Flanagan et al., 2021) which may suggest epigenetic silencing takes place prior to tumour formation, possibly confounding the premise of further epigenetic silencing in cancer tissue.

Non-canonical pathways are also dysregulated in colorectal cancer, with high expression of Wnt11a or ROR1 associated with poor prognosis (Nishioka et al., 2013; Zhou et al., 2017). Non-canonical Wnt signaling has been linked to proliferation, invasion, survival and metastasis of cancer stems cells (Katoh, 2017; Nishioka et al., 2013), although it should be noted that the role of non-canonical Wnts is context dependent. In certain situations, non-canonical Wnt5a has shown to be tumor suppressive and promote differentiation, most likely via cross-inhibition of canonical Wnt signaling (Zhou, Kipps, & Zhang, 2017).

## 4 Wnt pathway dysregulation in pancreatic cancer

*RNF43* mutations are the most common Wnt pathway mutations in pancreatic cancer, observed in 5-10% of cases (Fig. 2) (Bailey et al., 2016; Tu et al., 2019; Witkiewicz et al., 2015). Genetic lesions in *APC* or *AXIN1/2* are less common, observed in 1-5% of cases. As expected, *RNF43*-mutant pancreatic cancer xenografts remained dependent on Wnt ligand. However, a significant proportion of organoids derived from PDAC patients with wild-type *RNF43* also remained dependent on Wnt ligands, suggesting opportunities for Porcupine inhibitors and other ligand-dependent therapeutics exist beyond *RNF43* mutations in pancreatic cancers (Seino et al., 2018).

At the expression level, upregulation of β-catenin, Wnts and Frizzled receptors was seen in a gene-array analysis of pancreatic ductal adenocarcinomas (Pasca Di Magliano et al., 2007) and elevated nuclear β-catenin is associated with high grade neoplasms and poor prognosis (Al-Aynati, Radulovich, Riddell, & Tsao, 2004; Sano et al., 2016). High epithelial expression of Wnts in pancreatic tumors was also associated with poor prognosis (Seino et al., 2018). Ectopic expression of Wnt1 or β-catenin accelerated tumor growth and decreased survival in a murine pancreatic cancer model (Sano et al., 2016).

Wnt5a is elevated in pancreatic cancer tissue versus surrounding non-cancerous tissues and promotes invasion and proliferation whilst inhibiting apoptosis. Although typically considered a non-canonical Wnt, these effects appear to be via β-catenin signaling (Bo, Gao, Chen, Zhang, & Zhu, 2016; Griesmann et al., 2013; Ripka et al., 2007). The non-canonical pathway receptor ROR1 is overexpressed in circulating tumor cells from pancreatic cancer patients and knockdown of ROR1 reduces invasiveness (Xu, Shen, Xu, Wang, & Ni, 2018). Consistent with non-canonical signaling playing a role in pancreatic cancer, ROR2 expression in tumor epithelium and stroma is associated with poor prognosis in this indication (J. Huang et al., 2015).

## 5 Wnt pathway dysregulation in gastric cancer

Gastric cancers have one of the highest incidence of Wnt pathway mutations among cancers of the digestive tract (Flanagan, Vincan, & Phesse, 2017). *CTNNB1* mutations occur in up to 26% of gastric cancers and are associated with nuclear localization of β-catenin (Clements et al., 2002). Several *CTNNB1* single-nucleotide polymorphisms are associated with gastric cancer susceptibility and prognosis (Wang et al., 2012). *APC* and *AXIN1/2* mutations also occur in 5-10% of patients, although prevalence of *APC* mutations has been reported to be as high as 25% in some studies (Z. Fang et al., 2012; Horii et al., 1992; Pan, Liu, Zhang, You, & Lu, 2008). A significant proportion of gastric cancers contain ligand-dependent aberrations. *RNF43* mutations occur in 10% of patients, rising to 54% of MSI tumors, a high proportion of which are the aforementioned G659Vfs*41 frameshift truncations (Wang et al., 2014). *RSPO2* fusions have also been reported in gastric tumor xenografts (Li et al., 2018), although prevalence data is not available. Interestingly, patient-derived gastric tumor organoids mimic the variety of Wnt-pathway mutations observed in the clinic and reveal novel mutational and methylation patterns (CpG island methylation phenotype (CIMP^+^); DNA methylation) to achieve Wnt and/or RSPO independence. Importantly, organoids independent of RSPO (typically *RNF43* loss of function mutants) remain sensitive to Wnt inhibition (Nanki et al., 2018).

Beyond mutations, gene expression analysis found 46% of gastric cancers had upregulation of canonical and non-canonical Wnts, or their downstream pathway components (Ooi, Ivanova, Wu, Lee, & Tan, 2009). Conversely, multiple SFRPs and Dkks are downregulated by promoter hypermethylation (Chiurillo, 2015). Of note, Frizzled7 and Wnt5a are highly expressed in gastric cancer and both are associated with poor outcomes (Li et al., 2018; Maeda et al., 2020). Frizzled7 was identified as the predominant receptor responsible for transmitting Wnt signaling in human gastric cancer cells and genetic deletion of Frizzled7 prevented growth of gastric adenomas with or without *APC* mutations (Flanagan et al., 2019). Inhibiting Wnt5a blocked fibroblast-induced gastric cancer cell proliferation and migration *in vitro,* and metastasis of gastric cancers *in vivo* (Hanaki et al., 2012; Kurayoshi et al., 2006; Maeda et al., 2020). Together, these observations suggest that, even in the presence of downstream Wnt mutations, upstream inhibitors including those targeting Porcupine could have utility in gastric cancer.

## 6 Wnt pathway dysregulation in biliary cancer (Cholangiocarcinoma)

Only a small proportion of bile duct cancers carry genetic mutations in Wnt pathway components. *RNF43* mutations were observed in 1-2% of patients (Weinberg et al., 2019), although higher rates (4-9% have been seen in Asian cohorts, particular those associated with liver fluke infection (Chan-On et al., 2013; Ong et al., 2012). However, high expression of stromal Wnt7b and Wnt10a, and upregulation of several Frizzled receptors has been observed (Boulter et al., 2015), with greater than 75% of patient samples in another study showing positive expression of Wnt3a, Wnt5a and Wnt7b (Loilome et al., 2014). In addition Boulter and colleagues demonstrated efficacy in a biliary tract preclinical model with C-59, a Porcupine inhibitor, demonstrating that Wnt ligand is required for proliferation of these tumours (Boulter et al., 2015). Hypermethylation induced silencing of negative pathway regulators such as *SFRP2* and *DKK2* has also been observed (Goeppert et al., 2014). These data are suggestive of highly activated extracellular Wnt signaling in biliary cancers, which may therefore be suitable targets for inhibitors of Porcupine.

## 7 Wnt pathway dysregulation in hepatocellular carcinoma (HCC)

*CTNNB1* and *AXIN1/2* mutations occur in a significant proportion of HCC patients (Miyoshi et al., 1998; Schulze et al., 2015; Taniguchi et al., 2002), and mice with liver-targeted disruption of *APC* or overexpression of an oncogenic form of β-catenin develop hepatic tumors indicating a role for downstream canonical Wnt pathway activation (Colnot et al., 2004; de La Coste et al., 1998). Another study showed that partial ablation of APC also induced liver tumorigenesis in mice (Buchert, Athineos, Abud, Burke, & Faux, 2010).

In contrast, *ZNRF3* mutations were observed in only 3% of HCC (Schulze et al., 2015) and *RSPO* fusions are rare, although elevated RSPO2 was seen in a subset of HCC patients. Co-amplification of *RSPO2* and *MYC* has been observed in HCC, although the functional consequence of this is unclear (Sanchez-Vega et al., 2018). Elevated Wnt3/4/5a and suppression of SFRPs have also been observed, an expression pattern that did not correlate with *CTNNB1* mutations but was associated with loss of differentiation and cirrhosis, and activation of PCP pathways (Bengochea et al., 2008). Functional overexpression of RSPO2 with combined loss of transformation-related protein 53 (TP53) led to development of liver cancer in mice models (Conboy et al., 2019), implicating a role for extracellular Wnt ligands in liver tumorigenesis.

## 8 Pre-clinical evidence supporting use of porcupine inhibitors in gastrointestinal cancers

The pivotal role of Wnt signaling in gastrointestinal cancers has led to a search for therapeutics that target this pathway. A major challenge has been finding tractable targets, given the non-enzymatic nature of key intracellular components such as APC, AXIN and β-catenin. The majority of pathway inhibitors explored to date are antibody or biologicals-based approaches such as Frizzled decoy receptors and Frizzled blocking antibodies. Clinical studies of these therapeutics have been limited, hampered by toxicity issues (Table 1). A handful of small molecules have also been developed, such as CWP232291, which degrades β-catenin via an unknown mechanism (Pak et al., 2019), and PRI-724, which has been reported to block β-catenin/CBP interactions. (Table 1). However both compounds require intravenous dosing, and reported effects in the clinic to date are minimal, with no direct evidence of target engagement (El-Khoueiry et al., 2013; K. Kimura et al., 2017; Ko et al., 2016; Lee et al., 2020). Tankyrase inhibitors, which can antagonize Wnt signaling via Axin stabilisation, have failed to advance into the clinic due to lack of a therapeutic window versus gastrointestinal toxicity, as well as non-Wnt related effects (S.-M. A. Huang et al., 2009; Y. Zhong et al., 2016). Antibodies directed against Dkk-1, such as DKN-01 and BHQ880 are also being tested. Although Dkk-1 functions as a naturally occurring negative regulator of Wnt signaling, Dkk-1 expression is often associated with poor prognosis in gastrointestinal cancers, and Dkk-1 blocking antibodies can inhibit tumor growth in pre-clinical animal models (Kagey & He, 2017). Such effects may arise from the complexity of Wnt signaling; for example, Dkk-1 has been linked to promotion of metastasis via activation of the non-canonical PCP-pathway, and also to immune suppression. It is speculated that the role of Dkk1 may be altered in different contexts, including when the canonical Wnt pathway is constitutively activated (Kagey & He, 2017).

**Table 1:** Wnt Pathway Inhibitors in Cancer Clinical Trials

Drug Identifier	Mode of Action	Trial Identifier	Regimen	Cancer Indication	Trial Phase	Study Status or Clinical Results
OMP-54F28 (Ipafricept)	Fzd8-Fc Decoy receptor	NCT01608867	Monotherapy	Solid tumors	1	Dysgeusia and fragility fractures observed. Best response was SD (Jimeno et al., 2017)
		NCT02092363	In combination with paclitaxel and carboplatin	ovarian cancer;	1	ORR 76%, comparable with historical data. Development in ovarian stopped due to bone toxicity (Moore et al., 2019)
		NCT02069145	In combination with sorafenib (HCC)	Hepatocellular carcinoma	1	Study completed, no results reported
		NCT02050178	In combination with Gemcitabine and Nab-paclitaxel	1L pancreatic ductal adenocarcinoma	1	35% PR, 46% SD. Study terminated due to bone toxicity (Dotan et al., 2020)
OMP18R5 (Vantictumab)	Anti-Fzd7 antibody, also neutralizes binding to Wnts to Fzd 1, 2, 5,8	NCT01345201	Monotherapy	Solid tumors	1	Study completed, no results reported
		NCT01957007	In combination with docetaxel	Previously treated Non-small cell lung cancer;	1	Study completed, no results reported
		NCT02005315	In combination with Nab-Paclitaxel and Gemcitabine	1L pancreatic ductal adenocarcinoma	1	ORR 42%. Study terminated due to bone toxicity (Davis et al., 2020)
		NCT01973309	In combination with paclitaxel	Locally recurrent/ metastatic breast cancer	1	ORR 31%. Wnt pathway gene signature associated with longer PFS/OS. Bone fractures observed (Diamond et al., 2020)
		NCT04176016	monotherapy	Synovial sarcoma		Recruiting
OTSA101	Yttrium90 radiolabelled Anti-Fzd10 antibody	NCT01469975	monotherapy	Synovial sarcoma	1	Most common adverse events were hematologic disorders. SD in 3/8 patients (Giraudet et al., 2018)
		NCT04176016	monotherapy	Synovial sarcoma	1	Recruiting
OMP131R10	Anti-R-spondin3 antibody	NCT02482441	With or without FOLFIRI	*RSPO3* biomarker-positive metastatic colorectal cancer	1	Best response was SD in 3/7 patients treated with chemo combination (Bendell et al., 2016)
Foxy-5	Wnt5a mimetic	NCT02655952	monotherapy	Breast cancer; colorectal cancer; prostate cancer	1	Completed. No efficacy data reported
		NCT02020291	monotherapy	Breast cancer; colorectal cancer; prostate cancer	1	Completed. No efficacy data reported
		NCT03883802	As Neoadjuvant with surgery+FOLFOX	Wnt5a low colon cancer	2	Recruiting
PRI-724	Inhibitor of TCF-CBP interaction	NCT01302405	Monotherapy	Advanced solid cancers	1	No responses reported (El-Khoueiry et al., 2013)
		NCT01606579	Monotherapy or in combination with dasatinib, or cytarabine	Acute and chronic myelogenous leukaemia	1/2	Completed. No results reported
		NCT01764477	In combination with Gemcitabine	Advanced/metastatic pancreatic adenocarcinoma	1	Rest response was SD in 8/20 patients (Ko et al., 2016)
CWP232291	Induces degradation of beta catenin. Molecular target not defined	NCT01398462	Monotherapy	Acute Myeloid Leukaemia	1	ORR 3% GI side-effects were most common (Lee et al., 2020)
		NCT03055286	In combination with cytabarine	Acute Myeloid Leukaemia	1/2	Active, not recruiting
		NCT02426723	Monotherapy or in combination With Lenalidomide and Dexamethasone	Relapsed/refractory multiple myeloma	1	Completed. 4/14 patients with response, 4 had stable disease (Manasanch et al., 2017)
SM04755	Small molecule Wnt inhibitor	NCT02191761	Monotherapy	Advanced CRC, gastric, HCC and pancreatic cancer	1	Completed. No results reported
Cirmtuzumab	Anti-ROR1 antibody	NCT05156905	In combination with docetaxel	Metastatic castration resistant prostate cancer	2	Not yet recruiting

Source: Clinicaltrials.gov. Data collected on 10^th^ January 2022. Withdrawn trial or trials terminated early are not included. Dkk blocking antibodies such as DKN01 and BHQ880 were not included due to observed effects on other (non Wnt) pathways.

More recently, several small molecule drug candidates with attractive pharmacokinetic properties have been developed that target Porcupine (Table 2; (Bhamra et al., 2017; B. Chen et al., 2009; J. Jiang et al., 2018; Liu et al., 2013; Madan, Ke, et al., 2016; Proffitt et al., 2013)). Pre-clinical evidence suggests that Porcupine inhibitors exert anti-tumor effects via multiple mechanisms, and data from gastrointestinal models is summarised below.

**Table 2:** Porcupine Inhibitors in Cancer Clinical Trials

Drug Identifier	Trial identifier	Regimen	Cancer Indication	Clinical Phase
WNT974 (previously LGK974)	NCT02278133	In combination with LGX818 and Cetuximab	Metastatic colorectal cancer with *BRAFV600* mutations and *RSPO* fusions or *RNF43* mutations	Phase I/II
	NCT01351103	Monotherapy OR Combination with PDR001 (Spartalizumab)	Advanced solid tumors with Wnt pathway activation (monotherapy) Various advanced malignancies naïve or refractory to PD-L1 inhibitors (combination with PDR001)	Phase I
ETC-159	NCT02521844	Monotherapy OR Combination with Pembrolizumab or Denosumab	Advanced solid tumors	Phase I
CGX1321	NCT02675946	Monotherapy OR Combination with Pembrolizumab	Advanced solid tumors (monotherapy) Advanced colorectal cancer (combination)	Phase I
	NCT03507998	Monotherapy	Advanced GI tumors	Phase I
RXC004	NCT03447470	Monotherapy and in combination with Nivolumab	Advanced solid tumors	Phase I
	NCT04907851	Monotherapy	Advanced solid tumors	Phase II
	NCT04907539	Monotherapy and in combination with Nivolumab	RNF43/RSPO mutant microsatellite stable metastatic colorectal cancer	Phase II
XNW7201	NCT03901950	Monotherapy	Advanced solid tumors	Phase I

Source: Clinicaltrials.gov. Data collected on 10^th^ Jan 2022. Withdrawn trials not included

## 9 Effects of porcupine inhibitors on tumor growth and proliferation

Porcupine inhibitors have proven effective in inhibiting proliferation of cell lines and organoids derived from gastric, colorectal, pancreatic and biliary cancers. They also show potent inhibition of tumor xenograft growth in mice (Boulter et al., 2015; Li, Cao, et al., 2018; Madan, Ke, et al., 2016; Mo et al., 2013). Effects are limited to models derived from Wnt-dependent tumors, in particular those with *RSPO2/3* fusions or *RNF43* mutations, consistent with the role of Porcupine in Wnt secretion (X. Jiang et al., 2013; Koo et al., 2015; Woodcock et al., 2019). Inhibition of tumor proliferation was accompanied by downregulation of Wnt targets such as Axin2 and c-Myc, and loss of cell cycle and stem cell markers (Madan et al., 2016). In addition, Porcupine inhibitors reduce glucose uptake by cancer cells *in vitro* and *in vivo* (Phillips et al., 2021 and Phillips et al, manuscript submitted for publication) and several studies demonstrate elevated caspase 3/7 expression and induction of apoptosis (Bagheri, Tabatabae Far, Mirzaei, & Ghasemi, 2020; Boulter et al., 2015; Mo et al., 2013), all of which may contribute to inhibition of tumor growth. The multi-modal effects of Porcupine inhibitors may explain why established *RSPO2/3*-fusion tumours are rapidly cleared following treatment with the porcupine inhibitor LGK974, even though treatment had no effect on the normal growth of intestinal crypts (Han et al., 2017). Effects of Porcupine inhibitors are long-lasting, with no tumor regrowth observed within 6 weeks of treatment withdrawal (Madan, Ke, et al., 2016) and significantly reduced growth was seen when Porcupine-inhibitor treated tumors were reimplanted into naïve, untreated mice (Phillips et al., 2021 and Phillips et al, manuscript submitted for publication).

Most studies of Porcupine inhibitors have focused on tumor models with ligand-dependent Wnt pathway activation, under the assumption that downstream pathway activation such as that seen with mutations in APC and β-catenin would be unaffected by extracellular Wnt levels. Indeed, it has been reported that the Porcupine inhibitor LGK974 accelerates tumorigenesis in APC-deficient mice models by selectively blocking wild-type stem-cell proliferation, and thus favouring growth of APC-mutant cells (Huels et al., 2018). However, it has also been reported that Porcupine inhibitors could inhibit growth of a colon cancer cell line with mutations in *CTNNB1* (Voloshanenko et al., 2013). Other colon cancer cells lines, including some with *APC* mutations also appeared dependent on Wnt/Fzd signaling for growth (Vincan et al., 2007). Small molecule Porcupine inhibition in these cell lines resulted in decreased pathway activation, anti-proliferative effects and reduced cell viability. Taken together these data suggest patients with cancers bearing a subset of downstream mutations in the Wnt pathway, traditionally expected to be insensitive to Porcupine inhibitors, may well in fact receive therapeutic benefit from such treatment. However, further research is needed to determine which *APC/CTNNB1* mutation subtypes retain Wnt dependence, and the mechanism(s) involved.

## 10 Effect of porcupine inhibitors on cancer stem cells

Tumor recurrence following chemotherapy occurs through clonal replacement and reactivation of dormant cancer stem cells (CSCs) (Jahanban-Esfahlan et al., 2019). Canonical Wnt signaling prevents differentiation of CSC and promotes their retention to fuel tumor expansion by direct effects on CSCs, but also via CSC-stromal/immune interactions (Regan et al., 2017; Vermeulen et al., 2010). These findings go some way to reconcile the paradoxical observation that despite all cells within a tumor harbour Wnt-activating mutation, only a subfraction of cancer cells display CSC properties. Non-canonical Wnt signaling also promotes survival and resistance to therapy of CSCs through PI3K-AKT signaling activation (reviewed in Katoh, 2017). The Porcupine inhibitor LGK974 inhibited the ability of breast cancer stem cells to proliferate and form mammospheres (Zhao et al., 2014), and reduced the cancer stem cell compartment of established cutaneous squamous cell carcinomas in mice (Zimmerli et al., 2018). Mutation of *RNF43* or *ZNRF3* in mice intestines led to adenoma growth from LGR5-expressing stem cells, which was dependent on Wnt3 secreted by Paneth cells, and could be blocked by treatment with a Porcupine inhibitor (Koo et al., 2015). Acute treatment of mice with a high dose of a Porcupine inhibitor (i.e. higher doses than those which have shown anti-tumor effects) induced an initial burst of proliferation of intestinal stem cells as they converted into transit-amplifying cells, with a loss of stem cell self-renewal, supporting the critical function of Wnt signaling to maintain stemness and prevent differentiation in the gut (Kabiri et al., 2018). Consistent with this, Porcupine inhibitors have shown a striking ability to promote epithelial differentiation within the tumor, most notably by upregulation of mucins (X. Jiang et al., 2013; Madan, Ke, et al., 2016).

## 11 Effect of porcupine inhibitors on invasion and metastasis

Epithelial-mesenchymal transition (EMT) has been linked to invasion and metastasis in intestinal cancers. Reactivation of key developmental pathways, including the Wnt pathway, leads to loss of epithelial architecture and disruption of the basement membrane, which enables cancer cells to invade healthy tissues and migrate into the lymphatic system or bloodstream, leading to metastasis. Canonical and non-canonical PCP Wnt pathways have been implicated in CRC cell migration (Al-Aynati et al., 2004; Katoh, 2017; Y. Zhang et al., 2016; Z. Zhang, Wang, Zhang, Zhong, & Yang, 2018). The Porcupine inhibitor IWP-2 was shown to reduce migration and invasion of a gastric cancer cell line (Mo et al., 2013), and other Porcupine inhibitors reduced invasion of renal cancer cells *in vivo* (Li et al., 2020 and prolonged metastasis-free survival in an *in vivo* model of Ewing sarcoma (Hayashi et al., 2017). Effects of Porcupine inhibitors on migration *in vivo* may be due to direct effects on cancer cells and/or cell-nonautonomous effects related to interactions with neighboring stromal tissues.

## 12 Effects of porcupine inhibitors on fibrosis

Another corollary of EMT is the induction of fibrosis. Wnt signaling has long been implicated in fibrosis (Akhmetshina et al., 2012) and stromal signaling plays a key role in cancers, with growing evidence that cancer-associated fibroblasts (CAFs) play a role in immunosuppression as well as remodelling (Hilmi, Nicolle, Bousquet, & Neuzillet, 2020). Wnt5a functions as a supportive niche factor in gastric cancer, where it is upregulated by the tumor microenvironment (Hayakawa et al., 2015; Maeda et al., 2020). Wnt5a is also associated with the strong desmoplastic reaction observed in pancreatic cancer (Pilarsky et al., 2008). In colorectal cancers, upregulation of endogenous RSPO2/3 was strongly associated with a defined CAF signature and with the CMS4 (mesenchymal) consensus molecular subtype (Kleeman et al., 2020). Although data on fibrosis is lacking from cancer models, Porcupine inhibitors have demonstrated profound anti-fibrotic effects in models of skin, lung and kidney fibrosis (Bunyard et al., 2019; C.-W. Chen et al., 2017; Madan, Patel, et al., 2016).

## 13 Reversal of immune evasion by porcupine inhibition

Immune evasion plays a key role in tumor development. The presence of Wnt ligands in the microenvironment in gastrointestinal tumors positions them perfectly to impact the local tumor immune response. There is strong association between upregulation of Wnt signaling and downregulation of immune markers in cancers, including those of the gastrointestinal tract (Luke et al., 2019; Spranger, Bao, & Gajewski, 2015). Tumors may be classed as “hot” (i.e. inflamed), or “cold”, the latter state characterized by a tumor microenvironment lacking infiltrating lymphocytes and with high levels of suppressive regulatory T-cells (Ochoa de Olza, Navarro Rodrigo, Zimmermann, & Coukos, 2020). Cold tumors do not respond to immunotherapy agents. Of note, several canonical and non-canonical Wnt ligands and Frizzled receptors were upregulated in melanoma patients that failed to respond to anti-PD-1 therapy. The Porcupine inhibitor C59 reversed the ability of melanoma cancer cell-conditioned media to induce generation of dendritic cell-mediated regulatory-T cells *in vitro* (DeVito et al., 2021). In the immune cold B16F10 (C57BL/6 mice) melanoma tumor model, anti-PD-1 therapy was not effective, but treatment with the Porcupine inhibitor RXC004 was able to reduce tumor size both as a monotherapy and in combination with an anti-PD-1 inhibitor. Flow cytometry showed that the RXC004 treatment reduced the myeloid derived suppressor cells in the tumor microenvironment (Phillips et al., 2019, Phillips et al, manuscript submitted for publication). A combination of the Porcupine inhibitor C59 with anti-CTLA4 antibodies was also shown to have synergistic anti-tumor effects in the same model (Holtzhausen et al., 2015). These data suggest that Porcupine inhibitors may be effective in combination with immunotherapy in patients that currently do not respond to such therapies.

Further evidence that Porcupine inhibitors can reverse Wnt pathway-induced immune evasion comes from the immune hot CT26/BALBc colorectal syngeneic model, where RXC004 in combination with anti-PD-1 antibody resulted in a significant increase in the cytotoxic to regulatory T-cell ratio within the tumor. This was not seen in either monotherapy treatment arm (Phillips et al., 2019, Phillips et al, manuscript submitted for publication). The ability to reverse Wnt-ligand induced immune evasion in the wider tumor microenvironment may provide an explanation for the observed anti-tumor effects of Porcupine inhibitors even in cases where Wnt pathway is activated by downstream mutations within the tumor cell.

## 14 Potential toxicities of porcupine inhibitors identified *in vivo*

Wnt ligands play a vital homeostatic role in stem cell renewal, which means targeting the pathway has the potential for adverse effects, particularly within the intestinal epithelium. Ectopic expression of the negative Wnt regulator Dkk1 in mice caused loss of intestinal architecture (Kuhnert et al., 2004; Pinto, Gregorieff, Begthel, & Clevers, 2003), and high doses of Porcupine inhibitors have adverse effects on stem cell renewal with associated disruption of villi architecture (Kabiri et al., 2018). However, anti-tumor effects of Porcupine inhibitors have been observed at doses that have no effect on the normal intestine (Koo et al., 2015; J. Liu et al., 2013; Phillips et al, manuscript submitted for publication), suggesting there may be a safe therapeutic window for these compounds. The effects of Dkk1 overexpression on intestinal architecture were reversible, consistent with other studies that indicate the intestine has the ability to regenerate after insult (Kuhnert et al., 2004). A possible explanation for this may be redundancy in the stem cell populations, with HOPX-expressing stem cells, typically located higher in the intestinal crypt, able to compensate for loss of LGR5 expressing stem cells at the base of crypts, and vice-versa (Takeda et al., 2011), although such plasticity may be a relatively short-lived response to injury. Stem cells located at the intestinal crypt base are exposed to the highest levels of Wnt ligand provided by the niche (Paneth cells and stromal cells), and therefore may be more protected from drug-induced reduction of Wnt secretion (Kosinski et al., 2007). Consistent with this, mice with genetic ablation of *PORCN* in intestinal epithelial cells retained normal intestinal homeostasis due to compensatory Wnt secretion from neighboring stromal cells (Kabiri et al., 2014). Furthermore, pericryptal stromal cells have high expression of drug transporters, protecting the stem cell niche from xenobiotics, including Porcupine inhibitors (Chee et al., 2018).

Other *in vivo* toxicities observed with Porcupine inhibitors include loss of bone volume and density, most likely due to effects on osteoclasts. However, these effects could be mitigated by prophylactic treatment with bisphosphonates (Madan et al., 2018).

## 15 Porcupine inhibitors in the clinic

To date, five Porcupine inhibitors have entered phase I/II clinical trials (Table 2) in patients with advanced solid tumors. In NCT02521844, a maximum tolerated dose of 30 mg for ETC-159 (previously known as ETC-1922159) was established, limited by high bone marker turnover and compression fractures. Bone effects at lower concentrations were mitigated by prophylactic treatment with denosumab. Dysgeusia (loss or change in taste) was common, even at lower doses. Target engagement was demonstrated by inhibition of Axin2 expression in hair follicles and increased infiltration of T-cells into the tumor microenvironment. There were no objective responses, although 17% (2/12) patients had stable disease (Tan et al., 2018).

The WNT974 (previously LGK974) monotherapy dose-finding study did not find a maximum tolerated dose. Dysgeusia was the most common side effect, although bone effects were also observed in a subset of patients. Target engagement was demonstrated by reduction of Axin2 expression in skin. There were no objective responses;16% of 98 patients had stable disease (Rodon et al., 2021). In this study, 28 patients were genetically selected for Wnt pathway activation as part of a dose expansion arm, the majority of which had *RNF43* mutations. Of these 28 patients, 10 patients had stable disease. This result has led to others concluding that monotherapy Porcupine inhibitor treatment failed even with this enrichment for upstream Wnt pathway aberrations. However, since this dose expansion arm was run in 2015, knowledge of exactly which *RNF43* mutations result in loss of function has increased (Spit et al., 2020; Tu et al., 2019; Yu et al., 2020). With our current improved understanding, and excluding patients with co-occuring downstream Wnt pathway mutations, only 8 of these patients would now be considered to have RNF43 loss of function mutations. Of these 8 patients 7 had a best tumor response between -27 and +20%, suggesting a stringent patient selection strategy is likely needed in the clinic for Porcupine inhibitors (Rodon et al., 2021). When WNT974 was paired with the anti-PD-1 antibody spartalizumab, dose-limiting toxicities were reported in 2 patients, including one spinal compression fracture. One patient (4%) with triple-negative breast cancer had a partial response, 11 patients (41%) had stable disease, 13 patients (48%) had progressive disease. 75% of patients experienced a treatment-related adverse events, with dysgeusia and osteopenia among the most common (Janku et al., 2020).

Preliminary data from NCT03447470, a dose finding study for RXC004 in solid tumors, has recently been reported. The most common treatment related adverse events were fatigue, nausea, dysgeusia, vomiting and anorexia. No grade 4/5 adverse events or bone fragility events were reported, with patients receiving prophylactic denosumab. Five patients, all with Wnt pathway activated tumors, had stable disease, including one patient with biliary tract cancer, and two CRC patients with either an *RNF43* mutation or a *RSPO* fusion (Cook et al., 2021).

These early clinical studies have identified bone loss and dysgeusia as class effects of Porcupine inhibitors. The latter likely arises because Wnt signaling has been shown to be vital for proliferation of taste-progenitors and formation of taste buds (Gaillard et al., 2017; Prochazkova et al., 2017). Prophylactic treatment with bisphosphonates or denosumab has been shown to mitigate increases in bone turnover markers (Cook et al., 2021; Tan et al., 2018). Encouragingly, gastrointestinal side effects appear to be limited and manageable. Furthermore, the limited efficacy observed to date may be attributable to the studies being performed in heavily-pretreated patients with poor prognosis and lack of appropriate patient selection.

## 16 Conclusions and future perspectives

**Fig. 3:**
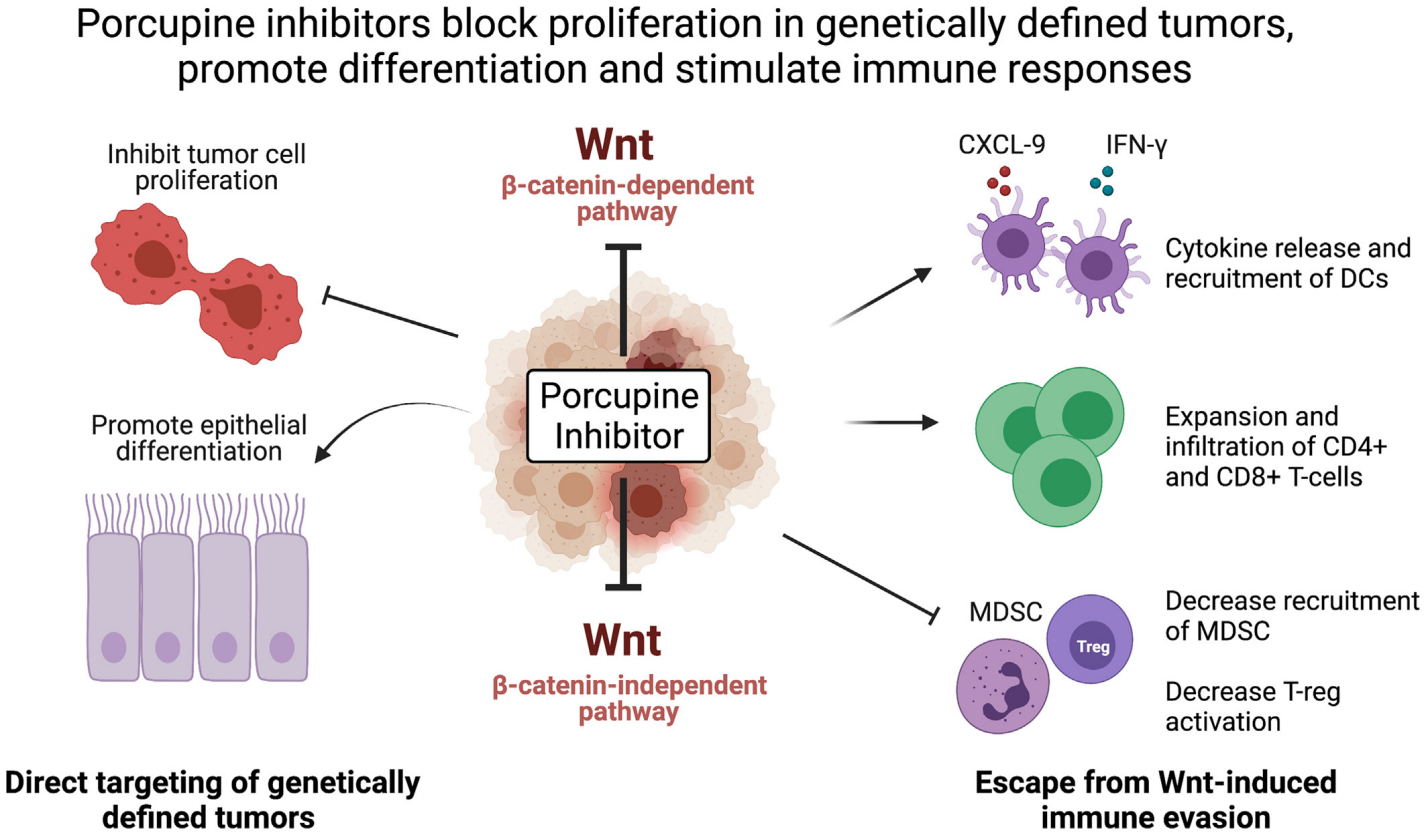
Porcupine Inhibitors Have Multiple Effects on Tumor Biology DC: Dendritic cells, MDSC: myeloid-derived suppressor cells. T-reg: regulatory T-cells. Image created with BioRender.com.

Porcupine inhibitors have the potential for multiple potentially beneficial effects on tumor biology (Fig. 3). There is substantial preclinical data supporting the use of Porcupine inhibitors in genetically-defined populations of patients with gastrointestinal cancers. Functionally relevant mutations are most common in CRC and pancreatic cancers. Patients with *RNF43* or *ZNRF3* truncations may be amenable to treatment and mutations in *RNF43* are particularly common in MSI high CRC and pancreatic cancers, although it should be noted that the majority of *RNF43* mutations found in MSI disease are the hotspot G659Vfs*41 frameshift, which has been reported to encode functional protein and may be simply a passenger mutation in this context (Tu et al., 2019). Tumors with *RSPO2/3* fusion and RSPO3 stromal overexpression are also likely to be susceptible to Porcupine inhibition, an intriguing prospect for CMS4 CRC subtype patients, where RSPO3 overexpression is common. There is also data to suggest Porcupine inhibitors may be effective in patients where Wnt signaling is activated by non-genetic/epigenetic mechanisms, and in certain patients with downstream Wnt pathway aberrations. However further work is needed to understand the processes involved, and to develop biomarkers to robustly identify such patients.

The effects of Porcupine inhibitors in reversing immune evasion provide a strong rationale for their use in combination with checkpoint inhibitors, particularly in patients previously unresponsive to anti-PD-1/PD-L1 therapy. MSI high CRC patients are currently indicated for anti-PD-1 therapy and exploration of anti-PD-(L)1/Porcupine inhibitor combinations in these patients may also be warranted. Similarly, Porcupine and BRAF inhibitors may be an attractive combination in CRC patients with both *BRAF* and *RNF43* mutations, particularly in light of the recent data from the BEACON study which demonstrated benefits of combining the BRAF inhibitor encorafenib with cetuximab in this setting (Tabernero et al., 2021). Additional combination opportunities are being explored pre-clinically, with synergy observed between ETC-159 and PI3K inhibitors or ETC-159 and PARP inhibition in Wnt-driven cancer models (Kaur et al., 2021; Z. Zhong et al., 2019).

Given the role of Wnt pathway in CSC maintenance, combination of Porcupine inhibitors with chemotherapy in selected patients may provide an interesting avenue of research, based on the rationale that Porcupine inhibitors may overcome chemoresistance.

Initial clinical data is promising in that, despite the homeostatic role of Wnt in the digestive tract, gastrointestinal side-effects at doses that engage the target are limited, indicating the presence of a therapeutic window. Effects on bone turnover can be mitigated with Receptor activator of nuclear factor-κB ligand (RANKL) inhibitors or bisphosphonate treatment, although the additional impact on taste may need to be managed in order to minimize associated weight loss. Since future strategies may include combining Porcupine inhibitors with other therapeutics, the potential for combined toxicities will also need to be managed. However initial data from NCT01351103, where WNT974 was used in combination with anti-PD-1 therapy showed the combination to be well-tolerated (Janku et al., 2020).

Conclusive clinical efficacy data on Porcupine inhibitors is currently lacking, with studies to date limited to small numbers of late stage, previously-treated patients with relevant somatic mutations. Key questions remain concerning optimal patient selection strategies for Porcupine inhibitors. Different approaches are being explored, including prospectively targeting patients with *RSPO2/3* fusions and *RNF43/ZNRF3* mutations with monotherapy, but also broader populations. WNT974 is under investigation in combination with anti-PD-1 in patients refractory to prior anti-PD-1 therapy, as well as other targeted populations. It is also interesting to note differences in dosing strategies between various Porcupine inhibitors; In NCT01351103, WNT974 was given as a priming dose when used in combination with anti- PD-1, RXC004 is dosed one daily, and ETC-159 demonstrated interpatient variability in pharmacokinetics and was dosed every other day. The impact of distinct dosing regimens and patient selection strategies is yet to be determined. Large scale, randomized, controlled clinical trials with robust biomarker assessments will be needed to elucidate whether Porcupine inhibitors will prove useful in gastrointestinal cancers, and to identify which patients are most likely to benefit from such treatments.

## Declaration of Competing Interest

S.W, C.E and CP are current employees of Redx Pharma PLC and hold shares or share options in Redx Pharma PLC. D.J.F. and O.J.S have no conflict of interest to declare.
